# Transfer of *Erwinia toletana* and *Erwinia iniecta* to a novel genus *Winslowiella* gen. nov. as *Winslowiella toletana* comb. nov. and *Winslowiella iniecta* comb. nov. and description of *Winslowiella arboricola* sp. nov., isolated from bleeding cankers on broadleaf hosts

**DOI:** 10.3389/fmicb.2022.1063107

**Published:** 2022-11-17

**Authors:** Carrie Brady, Sundeep Kaur, Bridget Crampton, Daniel Maddock, Dawn Arnold, Sandra Denman

**Affiliations:** ^1^Centre for Research in Bioscience, College of Health, Science and Society, University of the West of England, Bristol, United Kingdom; ^2^Centre for Ecosystems, Society and Biosecurity, Forest Research, Farnham, United Kingdom; ^3^Harper Adams University, Newport, United Kingdom

**Keywords:** *Erwinia*, *Erwiniaceae*, *Erwinia toletana*, bleeding canker, *Tilia*, *Platanus x acerifolia*, *Erwinia iniecta*, *Winslowiella*

## Abstract

Following a screening campaign of bleeding cankers of broadleaf hosts in Great Britain, numerous bacterial strains were isolated, identified by 16S rRNA and protein-coding gene sequencing and ultimately classified. During the course of the study, several Gram-negative, facultatively anaerobic strains were isolated from bleeding *Platanus* x *acerifolia* (London plane) and *Tilia* x *europaea* (common lime) cankers that could not be assigned to an existing species. Partial 16S rRNA gene sequencing placed these strains in the genus *Erwinia*, as a close phylogenetic relative of *Erwinia toletana*. In an effort to determine the taxonomic position of the strains, a polyphasic approach was followed including genotypic, genomic, phenotypic, and chemotaxonomic assays. Multilocus sequence analysis based on four protein-coding genes (*gyrB*, *rpoB*, *infB*, and *atpD*) confirmed the phylogenetic position of the strains as a novel taxon of subgroup 3 of the genus *Erwinia*, along with *E. toletana* and *E. iniecta*, and furthermore, provided support for their reclassification in a novel genus. Whole genome comparisons allowed the delimitation of the novel species and also supported the proposed transfer of subgroup 3 species to a novel genus in the *Erwiniaeae*. Phenotypically the novel species could be differentiated from *E. toletana* and *E. iniecta*, and the novel genus could be differentiated from the closely related genera *Erwinia* and *Mixta*. Therefore, we propose (1) the reclassification of *E. toletana* and *E. iniecta* in a novel genus, *Winslowiella* gen. nov., as *Winslowiella toletana* comb. nov. and *Winslowiella iniecta* comb. nov., with *W. toletana* comb. nov. as the type species (type strain A37^T^ = CFBP 6631^T^ = ATCC 700880^T^ = CECT 5263^T^), and (2) classification of the novel strains as *Winslowiella arboricola* sp. nov. (type strain BAC 15a-03b^T^ = LMG 32576^T^ = NCPPB 4696^T^).

## Introduction

The genus *Erwinia* has long been associated with plants and disease as either epiphytes, saprophytes, or pathogens (Hauben and Swings, 2015). Originally described in 1920 to unite the Gram-negative, fermentative, non-sporulating, peritrichously flagellated phytopathogenic bacteria, *Erwinia* is perhaps one of the oldest known plant-associated genera in the order *Enterobacterales* (Winslow et al., 1920). Indeed, numerous species in the genera *Brenneria*, *Enterobacter*, *Lonsdalea*, *Pantoea*, and *Pectobacterium* resided within *Erwinia* at some point in their taxonomic history. The genus currently comprises 21 species, including the important plant pathogens *Erwinia amylovora*, *Erwinia piriflorinigrans*, *Erwinia psidii*, *Erwinia pyrifoliae*, and *Erwinia uzenensis* as well the epiphytes *Erwinia billingiae* and *Erwinia tasmaniensis* (Parte et al., 2020). *Erwinia toletana* is considered an endophyte but this species plays an important role in olive knot formation through cooperation with the pathogen *Pseudomonas savastanoi* pv. *savastanoi* (Buonaurio et al., 2015).

Diseases typically caused by *Erwinia* species include fireblight of plants belonging to the family *Rosaceae*, particularly fruit trees (Vanneste, 2008); necrosis of pear blossom (López et al., 2011); dieback of *Psidium guajava* (Rodrigues Neto et al., 1987) and *Carica papaya* (Maktar et al., 2008); wilt and dieback of *Eucalyptus* species (Coutinho et al., 2011); and necrosis (Kim et al., 1996) and bacterial black shoot of pear trees (Matsuura et al., 2012). Apart from the diseases caused on fruit trees, there are few reports of *Erwinia* species isolated from woody hosts, specifically from broadleaf hosts.

As part of an ongoing survey of bleeding cankers and wetwood of broadleaf hosts in Great Britain,^1^ *Tilia* x *europaea* (common lime), *Platanus* x *acerifolia* (London plane), *Fagus sylvatica* (common beech), *Betula* spp. (birch), and *Populus* spp. (poplar) exhibiting symptoms were screened for the presence of bacterial pathogens. Following the isolation and identification of bacteria from bleeding cankers of these hosts, several strains from London plane and common lime could not be assigned to an existing species. Partial 16S rRNA gene sequencing of the strains demonstrated ∼99% similarity to *E. toletana*, but phylogenetically they appeared to belong to a novel taxon. A recent study examining the phylogenomic relationships between species of the family *Erwiniaceae*, identified three subgroups of species in the genus *Erwinia* (Soutar and Stavrinides, 2022). Subgroup 3 contained *E. toletana* and *Erwinia iniecta* and it was suggested that these species should be assigned to a novel genus. A second study demonstrated the close relationship between “*Pantoea beijingensis*,” *E*. *toletana* and *E. iniecta* and concluded that “*P. beijingensis*” should be renamed as “*Erwinia beijingensis*” (Xu et al., 2021). However, these suggestions were not formally proposed and implemented. A polyphasic approach was followed to determine the taxonomic position of the bleeding canker strains as a novel species within a novel genus, for which the name *Winslowiella arboricola* sp. nov. is proposed, as well as the reclassification of *E. toletana* and *E. iniecta* as *Winslowiella toletana* comb. nov. and *Winslowiella iniecta* comb. nov.

## Materials and methods

### Isolation and ecology

Bacterial strains were isolated from the bleeding cankers of *Platanus* x *acerifolia* (London plane) at St James Park, London and *Tilia* x *europaea* (common lime) at Tidworth Garrison, Wiltshire. Swab samples of the weeping exudate were collected from the London plane in May and October, 2020. A small bark panel (3 × 3 cm) was taken from the bleeding common lime in August 2019.

Swabs were rehydrated in sterile 1/4 Ringers, the swab spread on Luria-Bertani (LB) agar (Oxoid) and Eosin Methylene Blue (EMB) agar (Merck) which were initially incubated at 35°C under anaerobic conditions for 4 days to promote growth of facultatively anaerobic bacteria. Bark tissue was removed from the panel with a scalpel to expose the developing lesion. Wood chips were cut around the advancing lesion front, plated on LB agar and incubated at 25°C for 7 days. Single colonies were obtained through re-streaking on LB agar and incubation at 25°C.

The strains used in this study are listed in Supplementary Table 1.

### Genotypic characterisation

Genomic DNA for genotypic characterisation was extracted using an alkali lysis method (Niemann et al., 1997) and stored at −20°C. Strains were initially screened and tentatively identified based on partial 16S rRNA gene sequences (using primer *pD), and the almost complete 16S rRNA gene sequence determined for the proposed type strain using the primers published by Coenye et al. (1999). Standard PCR conditions and cycles were used with an annealing temperature of 55°C. Multilocus sequence analysis (MLSA) based on partial sequences of four housekeeping genes (*gyrB*, *rpoB*, *infB*, and *atpD*) was performed on the strains tentatively identified as *Erwinia* sp. by partial 16S rRNA gene sequencing. MLSA PCR conditions, cycles and primers were applied as previously described (Brady et al., 2008), although with an updated version of the *gyrB* amplification primers: gyrB 01.2F (5′ TAARTT YGAYGAYAACTCBTAYAAACT 3′) and gyrB 02.2R (5′ CMCCYTCCACHARGTASAKTTC 3′). Consensus sequences for the 16S rRNA and housekeeping genes were generated in UGENE v38.1 (Okonechnikov et al., 2012) and aligned and trimmed in MEGA X (Kumar et al., 2018) to the following lengths: 16S rRNA—1346 bp, *gyrB*—742, *rpoB*—637, *infB*—615 bp, and *atpD*—642 bp. Maximum likelihood phylogenetic tree construction was performed on both datasets in PhyML 3.0 (Guindon et al., 2010) with 1,000 bootstrap replicates and automatic model selection by Smart Model Selection (Lefort et al., 2017).

To examine the genetic diversity between strains of the potential novel species, BOX PCR was performed using the primer and conditions previously published (Versalovic et al., 1994). Amplification products were separated on 1.5% agarose at 50 V for ∼3 h.

### Genome features

The whole genomes of strains BAC 15a-03b^T^ and Til 1, isolated from London plane and common lime respectively, were sequenced by MicrobesNG (Birmingham, UK). DNA was extracted by enzymatic cell lysis and purified with SPRI (Solid Phase Reversible Immobilisation) beads, before sequencing using the Illumina HiSeq platform. Adapters were trimmed from reads using Trimmomatic 0.30 with a sliding window quality cut-off of Q15 (Bolger et al., 2014), while *de novo* assembly was performed using SPAdes version 3.11.1 (Bankevich et al., 2012) and the resulting contigs annotated in the prokaryotic genome annotation pipeline (PGAP) (Tatusova et al., 2016). Average nucleotide identity (ANI) and average amino acid identity (AAI) were performed on the novel strains, along with validly published *Erwinia* species and “*E. beijingensis*,” using FastANI (Jain et al., 2018) and the AAI-matrix calculator from the Kostas Lab (Rodriguez-R and Konstantinidis, 2016), respectively. Additionally, pairwise percentage of conserved proteins (POCP) were calculated using the script pocp.rb (Hoelzer, 2020) which follows the approach by Qin et al. (2014).

Pairwise phylogenomic comparisons, between the novel species and current closest relatives from the genera *Erwinia* and *Mixta*, were carried out on the Type (Strain) Genome Server (TYGS) (Meier-Kolthoff and Göker, 2019) using Genome Blast Distance Phylogeny (GBDP). Accurate intergenomic distances were inferred under the algorithm “trimming” and distance formula *d*_5_ (Meier-Kolthoff et al., 2013), with 100 distance replicates each. The resulting intergenomic distances were used to infer a balanced minimum evolution tree with branch support *via* FASTME 2.1.6.1 including SPR postprocessing (Lefort et al., 2015) and 100 pseudo-bootstrap replicates. The tree was rooted at the midpoint (Farris, 1972).

### Phenotypic and chemotaxonomic characterisation

Colony morphology was observed following growth of strains on LB agar incubated at 30°C for 48 h. The temperature range for growth was determined on LB agar incubated at 4, 10, 25, 30, 37, and 41°C. Tolerance of the novel species to salt was tested in saline-free nutrient broth with NaCl added in increments of 1% w/v from 1 to 10%, while pH tolerance was tested in LB broth with the pH adjusted from 4 to 10 in increments of 1 as previously described (Brady et al., 2022). Catalase and oxidase activity were determined by bubble production in 3% v/v H_2_O_2_ and by staining with Kovács reagent (1% tetra-methyl-*p*-phenylenediamine dihydrochloride), respectively. Cell size, morphology and motility were examined using a light microscope and the CellSens software v 1.11 (Olympus Life Science, Tokyo, Japan) following growth in LB broth at 25°C for ∼6 h. The flagella arrangement was visualised by transmission electron microscopy (FEI Tecnai 12 120kV BioTwin Spirit TEM) following negative staining as previously published (Brady et al., 2022).

Commercial phenotypic assays, including API 20E, API 50 CHB/E (bioMérieux), and GEN III GN/GP microplates (Biolog), were performed on strains BAC 15a-03b^T^, BAC 1-01-01, Til 1, and Til 5 according to the manufacturer’s instructions. The type strain of *E. toletana* LMG 24162^T^ was included as a positive control. The API 20E galleries were scored after 24 h incubation at 37°C, while the API 50 CHB/E galleries and GEN III microplates were scored after 24 h incubation at 30°C, and again after 48 h. The fatty acid methyl ester (FAME) profile of the novel species was determined by Fera Science Ltd. (York, UK). Following the protocol based on the Sherlock Microbial Identification System Version 6.4 (MIDI Inc.), strains were cultivated on TSA at 28°C for 24 h prior to fatty acid extraction. The results obtained were compared against the library RTSBA6 6.21.

### Virulence gene identification

The protein annotations generated through PGAP for the whole genome sequences of BAC 15a-03b^T^, Til 1, *E. toletana* DAPP-PG 735, *E. iniecta* B120^T^, and “*E. beijingensis*” JZB2120001^T^ were queried through two databases to assess the pathogenic potential of their proteome. DIAMOND version v2.0.11.149 (Buchfink et al., 2021) was used to query genomes with the BlastP command against the Virulence Factor Database (VFDB) (Liu et al., 2022), accessed October 3, 2022. A query cut-off of 97% and a percentage identity equal or greater than 50 were used to ensure high sequence alignment identification by DIAMOND against the VFDB (Doonan et al., 2019). To assess the microbe-plant interaction potential of the four species, the annotations were queried using BlastP + HMMER Aligner/Mapper against the “plant bacterial only interaction factors” (PIFAR-Pred) database through the PLant-associated BActeria web resource (PLaBAse) (Martínez-García et al., 2016; Patz et al., 2021). For the identification of type III secretion system effectors (T3SS), open reading frames (ORFs) were first identified using orfipy and exported in fasta format (Singh and Wurtele, 2021). ORFs were then individually uploaded to the Effectidor web server for the prediction of T3SS effectors (Wagner et al., 2022).

## Results and discussion

### Genotypic characterisation

**FIGURE 1:**
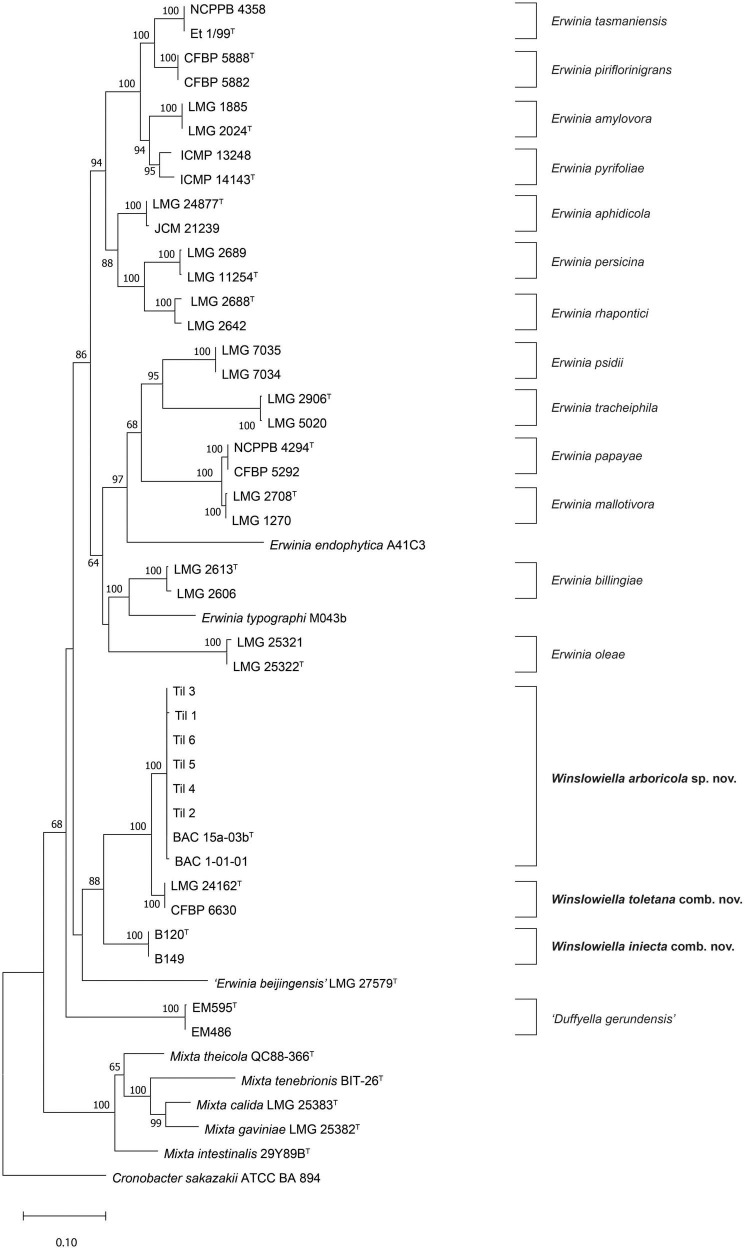
Maximum likelihood tree based on concatenated partial *gyrB*, *rpoB*, *atpD*, and *infB* gene sequences of *Winslowiella* gen. nov., *Winslowiella arboricola* sp. nov., and phylogenetic relatives. Bootstrap values after 1,000 replicates are expressed as percentages (values > 50% shown). *Cronobacter sakazakii* (ATCC-BAA 894) is included as an outgroup. The scale bar indicates the fraction of substitutions per site. ^T^, type strain.

Pairwise 16S rRNA sequence similarity of BAC 15a-03b^T^, the proposed type strain of the novel species, in EzBioCloud (Yoon et al., 2019) showed greatest similarity to *E. toletana* (99.4%), *Erwinia endophytica* (98.2%), and *E. billingiae* (97.9%). These similarity values are reflected in the 16S rRNA gene phylogenetic tree (Supplementary Figure 1) where BAC 15a-03b^T^ was situated on a separate branch between *E. toletana* and *E. endophytica*, albeit with no bootstrap support. The proposed novel species was confirmed as a new taxon in the concatenated MLSA phylogenetic tree (Figure 1), as all strains formed a single cluster without any reference strains of *Erwinia* species with validly published names, with 100% bootstrap support. This cluster was in a well-supported clade (88%) with *E. toletana* and *E. iniecta*, corresponding to Subgroup 3 from Soutar and Stavrinides (2022) providing support for their transfer to a novel genus. “*Erwinia beijingensis*” was situated on a separate branch on the border of this clade, but without bootstrap support, suggesting that its position could change with the addition of further novel species.

The BOX PCR profiles demonstrated that there were slight variations between strains isolated from the same tree host species, although identical clones isolated from common lime were also observed (Supplementary Figure 2). Several shared bands were observed between strains isolated from London plane and common lime. However, these are clearly different hosts situated in different counties, suggesting the strains do not originate from a single clone and that a genetically divergent population exists in Great Britain.

### Genome features

Assembly of the sequences for BAC 15a-03b^T^ and Til 1 resulted in genomes of 5.23–5.31 Mbp, with the DNA G + C content ranging from 53.5–53.6 mol%. Genome sequences were submitted to GenBank under the BioProject number PRJNA878947. Assembly accession numbers and genome features are listed in Supplementary Table 2. ANI values (Table 1) confirmed the status of the strains from bleeding cankers as a single novel taxon, with 100% similarity observed between the strains. The ANI data reflects the phylogenetic relationship observed between the novel species and *E. iniecta* and *E. toletana*, with similarity values ranging from 83.8 to 91.7% between the three species, although these are well below the suggested cut-off value of 95–96% for species delimitation (Goris et al., 2007). The remaining *Erwinia* species tested demonstrated lower values of 79.1–80.6% when compared to the novel species, *E. iniecta* and *E. toletana*. “*E. beijingensis*” exhibited ANI values of 80.4–80.5% to the three species under examination, which are in the same region as the values shared by *E*. *aphidicola* and the three species (80.5–80.6%). The conclusions inferred from the ANI analysis were confirmed by *in silico* DNA-DNA hybridisation (Meier-Kolthoff et al., 2013), the results for which are also presented in Table 1. The AAI data (Supplementary Table 3) were congruent with those from ANI, with higher values observed between the novel species, *E. iniecta* and *E. toletana* (86–95%) and lower values to the remaining *Erwinia* species (76–78%) providing justification for their transfer to a novel genus. The AAI values between the proposed novel genus and “*E. beijingensis*” are 81–82%, which are slightly higher than those exhibited by other *Erwinia* species but significantly less than the values observed within the novel genus. Genus delineation criteria, based on AAI, have been suggested with species exhibiting > 76% AAI to the type strain of the type species of the genus, and all type strains within the genus sharing > 74% AAI to each other (Nicholson et al., 2020). These cut-off values appear too relaxed for the *Erwiniaceae*, and Soutar and Stavrinides (2022) suggested that 79% may be an appropriate threshold for delineating genera within this family. However, several *Erwinia* species exhibited AAI values which can be considered “borderline.” The POCP values (Supplementary Table 3) demonstrated a similar pattern with higher values (75–87%) observed between species of the proposed novel genus, and lower values (64–73%) to “*E. beijingensis*” and *Erwinia* species. It is worth noting that *E. aphidicola*, *E. oleae*, and *E. persicina* all demonstrated higher POCP values to the proposed novel genus than “*E. beijingensis*.”

**TABLE 1:** DNA-DNA similarity values between *Winslowiella arboricola* sp. nov., *Winslowiella iniecta* comb. nov., *Winslowiella toletana* comb. nov., and existing species of the genus *Erwinia* based on average nucleotide identity (fastANI—lower left) and *in silico* DNA-DNA hybridisation (*is*DDH—upper right).

*is*DDH FastANI	1	2	3	4	5	6	7	8	9	10	11	12	13
1	100	100	26.3	45.3	22.1	21.5	21.5	20.8	21.0	21.0	20.6	21.4	21.3
2	100	100	26.3	45.3	22.1	21.5	21.6	20.8	21.0	21.0	20.7	21.4	21.3
3	83.8	83.9	100	26.2	22.5	21.4	21.8	20.9	21.2	21.1	20.7	21.5	21.3
4	91.7	91.7	83.9	100	22.0	21.4	21.4	21.8	21.0	21.0	20.5	21.3	21.3
5	80.4	80.4	80.5	100	100	21.4	20.7	20.6	20.5	20.5	20.5	21.2	21.1
6	79.8	79.9	79.7	79.8	79.0	100	24.1	21.0	23.3	30.0	20.7	40.9	29.4
7	80.5	80.5	80.6	80.6	79.4	81.9	100	21.5	24.7	23.9	20.8	24.5	24.3
8	79.6	79.6	79.6	80.3	78.6	79.4	80.2	100	20.8	20.8	21.0	21.4	21.0
9	79.9	79.8	79.8	79.8	79.2	81.1	83.2	79.4	100	23.2	20.5	23.8	23.3
10	79.7	79.7	79.7	79.9	79.0	85.6	81.7	79.2	81.1	100	20.5	32.3	37.7
11	79.1	79.1	79.3	79.1	78.9	79.2	79.4	79.4	79.3	79.3	100	20.7	20.5
12	79.7	79.7	79.9	79.9	79.2	90.8	82.1	79.4	81.4	86.9	79.1	100	31.2
13	79.8	79.9	79.7	80.0	79.4	85.3	81.9	79.6	81.0	89.4	79.2	86.2	100

Percentages above cut-off value for species delimitation (>95% for ANI and >70% for isDDH) are shaded. 1 = *Winslowiella arboricola* BAC 15a-03b^T^ (GCA_025527015), 2 = *Winslowiella arboricola* Til 1 (GCA_025527035), 3 = *Winslowiella iniecta* B120^T^ (GCA_001267535), 4 = *Winslowiella toletana* DAPP-PG 735 (GCA_000336255), 5 = “*Erwinia beijingensis*” LMG 27579^T^ (GCA_004022165), 6 = *Erwinia amylovora* ATCC 15580^T^ (GCA_017161565), 7 = *Erwinia aphidicola* JCM 21238^T^ (GCA_014773485), 8 = *Erwinia oleae* DAPP-PG531^T^ (GCA_000770305), 9 = *Erwinia persicina* NBRC 102418^T^ (GCA_001571305), 10 = *Erwinia piriflorinigrans* CFBP 5888^T^ (GCA_001050515), 11 = *Erwinia psidii* IBSBF 435^T^ (GCF_003846135), 12 = *Erwinia pyrifoliae* DSM 12163^T^ (GCA_000026985), 13 = *Erwinia tasmaniensis* ET1/99^T^ (GCA_000026185). ^T^, type strain.

**FIGURE 2:**
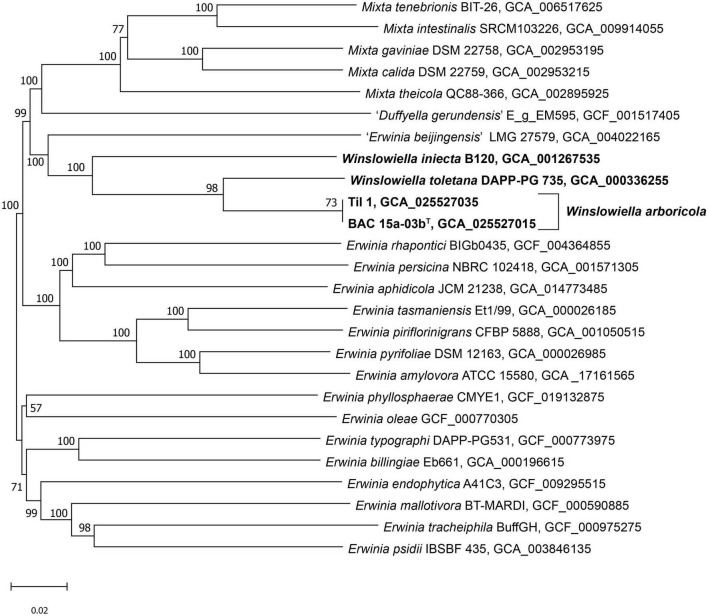
Phylogenomic tree of *Winslowiella* gen. nov., *Winslowiella arboricola* sp. nov., and phylogenetic relatives. GBDP pseudo-bootstrap support values > 60% shown at the nodes (from 100 replicates), with an average branch support of 85.4%. The branch lengths are scaled in terms of GBDP distance formula *d*_5_ and the tree is rooted at the midpoint. ^T^, type strain.

The phylogenomic tree generated in TYGS clustered the novel species, *E. toletana* and *E. iniecta* in a single clade on a separate lineage with 100% support (Figure 2). “*E. beijingensis*” was situated on a separate branch on the border of this clade, further removed from the proposed novel genus but with 100% support. The phylogeny of the whole genome tree demonstrates the divergence within the genus *Erwinia* with three clear subgroups of species, as defined previously (Soutar and Stavrinides, 2022) and confirms the proposal that *E. iniecta* and *E. toletana* should be reclassified within a novel genus along with the novel species.

### Phenotypic and chemotaxonomic characterisation

After 48 h growth on LB agar, colonies were round, 2–3 mm in diameter, smooth with entire edges, slightly convex, translucent, and cream to pale yellow in colour. Strains grew readily at temperatures of 10–30°C after incubation for 3 days, with weak growth observed at 4 and 37°C. Salt was tolerated by strains in broth supplemented with up to 7% NaCl, while growth in a narrow pH range of 6–8 was observed, with weak growth at pH 9. The proposed novel species is catalase positive and oxidase negative. Cells are straight rods with rounded ends, typically 0.6–1.3 × 1.5–3.5 μm in size, can occur singly, and in pairs or chains and motile by peritrichous flagella (Supplementary Figure 3).

The novel species could be differentiated from its current closest phylogenetic relatives by several phenotypic characteristics including reactions to acetoin production, tryptophan deaminase activity, aspartic acid and pectin (differentiation from *E. iniecta*) and L-rhamnose, amygdalin, β-methyl-D-glucoside and D-serine (differentiation from *E. toletana*). Differentiating phenotypic characteristics are presented in Table 2, and positive phenotypic characteristics shared by all current members of the proposed novel genus are listed in Supplementary Table 4. The fatty acid profile of the novel species was most similar to its current closest phylogenetic relative, *E. toletana*, with C_16:0_, C_18:1_ ω7*c*, and summed features 2 and 3 composing the major fatty acids. The complete fatty acid profiles of the novel *Erwinia* species and current closest phylogenetic relatives are listed in Table 3. “*E. beijingensis*” exhibited a distinctive fatty acid profile when compared to species of the proposed novel genus, with a high percentage of the cyclopropane fatty acid C_19:0_ ω8*c* and only a minor amount of summed feature 3, which is a major fatty acid of the novel genus. Phenotypic data available from literature for the genera *Erwinia* and *Mixta* (Liu et al., 2013; Hauben and Swings, 2015; Ramírez-Bahena et al., 2016; Rezzonico et al., 2016; Mavima et al., 2022) were examined for characteristics to support the differentiation of the proposed novel genus. Although complete phenotypic information for species of *Erwinia* and *Mixta* is not widely published (Palmer et al., 2018), some useful phenotypic traits are listed in Table 4.

**TABLE 2:** Phenotypic characteristics that can distinguish *Winslowiella arboricola* sp. nov. from its current closest phylogenetic relatives.

Characteristic	1	2	3	4
Acetoin production	−	−	+	+
Tryptophan deaminase	−	−	+	−
Nitrate reduction	−	−	+	nd
**Acid production from (API 50 CBH/E):**				
Sucrose	−	−	+	+
L-rhamnose	+	−	nd	+
Amygdalin	−	+	nd	nd
Glycogen	v	−	nd	nd
**Utilisation of (Biolog):**				
D-raffinose	v	+	+	−
β-methyl-D-glucoside	v*^a^*	−	+	+
D-salicin	v*^a^*	−	+	nd
*N*-acetyl-D-glucosamine	v*^a^*	−	+	+
3-methyl glucose	v	+	v	nd
L-fucose	v	+	−	+
L-rhamnose	+	−	+	−
L-aspartic acid	+	+	−	+
D-serine	−	+	−	+
Pectin	−	−	+	nd
Mucic acid	+	+	−	nd
Citric acid	+	+	−	+
Formic acid	v*^a^*	+	−	+
**Sensitivity to (Biolog):**				
Fusidic acid	v	−	−	nd
D-serine	v	+	−	nd

1 = *Winslowiella arboricola* (*n* = 3), 2 = *Winslowiella toletana* LMG 24162^T^, 3 = *Winslowiella iniecta* (*n* = 4), 4 = “*Erwinia beijingensis*” (*n* = 4). Data for 3 and 4 taken from the literature (Liu et al., 2013; Campillo et al., 2015). *n*, number of strains. +, 90–100% strains +; −, 91–100% strains −; v, variable; nd, not determined. ^*a*^Type strain negative.

**TABLE 3:** Major fatty acid composition (percentage of peak areas) of *Winslowiella arboricola* sp. nov. and current closest phylogenetic relatives.

Fatty acid	1	2	3	4
**Saturated fatty acids**				
C_12:0_	7.7 (±0.4)	9.2	–	4.6
C_16:0_	33.9 (±0.4)	29.7	32.0	26.8
C_17:0_	2.0 (±0.8)	2.4	–	1.9
**Unsaturated fatty acids**				
C_18:1_ ω7*c*	13.6 (±0.4)	8.5	18.8	22.8
**Cyclopropane fatty acids**				
C_17:0_	7.3 (±3.0)	14.0	10.3	3.0
C_19:0_ ω8*c*	–	–	–	16.3
**Summed features**				
2: C_14:0_ 3-OH and/or iso-C_16:1_	9.8 (±0.5)	12.2	–	10.5
3: C_16:1_ ω7*c* and/or C_16:1_ ω6*c*	21.3 (±3.2)	18.7	17.5	1.7

1 = *Winslowiella arboricola* (*n* = 3), 2 = *Winslowiella toletana* LMG 24162^T^, 3 = *Winslowiella iniecta* B120^T^, 4 = “*Erwinia beijingensis*” JZB2120001^T^. Values are expressed as the average if more than one strain per species were investigated, with the standard deviation shown in parentheses. Data for 2–4 taken from the literature (Liu et al., 2013; Campillo et al., 2015; Rezzonico et al., 2016). *n*, number of strains.

**TABLE 4:** Phenotypic characteristics for differentiation of the novel genus *Winslowiella* from the genera *Erwinia* and *Mixta*.

Characteristic	1	2	3
Growth at 40°C	−	v	+
Acetoin production	v	v	+
B -galactosidase	v	v	+
**Utilisation of (Biolog):**			
D-cellobiose	+	v	+
D-arabitol	+	v	−
D-galacturonic acid	+	v	v
D-glucuronic acid	+	v	nd
DNA G + C content (mol%)	51.1–53.6	49.8–54.1	56.1–59.1
Major fatty acids (>5%)	C_16:0_, C_18:1_ ω7*c*, summed feature 3	C_12:0_, C_16:0_, C_18:1_ ω7*c*, summed feature 2 and 3	C_14:0_, C_16:0_, summed features 2, 3 and 8

1 = *Winslowiella*, 2 = *Erwinia*, 3 = *Mixta*. Data from this study and the literature (Liu et al., 2013; Hauben and Swings, 2015; Ramírez-Bahena et al., 2016; Rezzonico et al., 2016; Mavima et al., 2022). +, 90–100% strains +; −, 91–100% strains −; v, variable; nd, not determined.

### Virulence gene identification

The BlastP alignment results from the VFDB using the query parameters revealed the presence of 101 virulence genes for BAC 15a-03b^T^, Til 1 and *E. toletana* DAPP-PG 735 while 121 and 105 alignments were identified in *E. iniecta* B120^T^ and “*E. beijingensis*” JZB2120001^T^, respectively. Within each comparison 11–23 type III secretion effector delivery system proteins such as SpaQ and Spa9 were identified, excluding “*E. beijingensis*.” This is most likely due to the stringent search parameters used, which when relaxed revealed a number of T3SS related genes which were previously identified (Xu et al., 2021). The other major protein roles identified were related to motility, immune modulation, invasion, and adherence. The presence of a T3SS has long been used to implicate bacteria as phytopathogens. Within the order Enterobacterales, it is usually an indicator of plant pathogens such as the soft rot pathogen *Pectobacterium atrosepticum* or the blight pathogens *E. amylovora* and *E. pyrifoliae* (Toth et al., 2006; Kube et al., 2010). Due to the importance of the secretion system, Effectidor was used to identify T3SS proteins and effectors from ORFs for each genome. Each species was found to contain 26 core TS33 proteins and related effectors, some of which appear to be novel.

The PIFAR-predictions also demonstrated similar implications for the novel species, with the identified “plant only interaction factors” being “genes that are related to plant interaction and virulence.” Nine different plant cell wall degrading enzymes (PCWDE) were identified in each genome. Many genes relating to detoxification and adhesion were identified amongst other plant interaction factors, such as hormone regulation and exopolysaccharides. Notable potential virulence genes are listed in Supplementary Table 5. The identified genes from both the VFDB and PIFAR comparisons indicate that all four species investigated contain genes used in invasion, adhesion and breakdown of plant tissue, indicating a strong pathogenic potential. Given the point of isolation of the novel species was from bleeding cankers of broadleaf hosts, further investigation of its role as novel pathogen is warranted.

## Conclusion

The genus *Erwinia* has long been a repository for species causing necroses, wilts and soft rot of plant hosts. However, the *Erwinia* species with true phytopathogenic potential are outweighed by those seldom-isolated species or those reported as epiphytes, isolated from insects or opportunistic human pathogens. Currently, 21 species with validly published names are assigned to *Erwinia* (Parte et al., 2020) with more than half of these described in the last two decades. The relative ease and lowering costs of whole genome sequencing has resulted in a wealth of *Erwinia* genomes available for analysis, allowing construction of stable phylogenomic analyses. An increase in the classification of novel species has revealed the intra-genus divergence within *Erwinia* (Soutar and Stavrinides, 2022), and it is becoming clear that *Erwinia* will require further taxonomic re-evaluation as more novel species are described. The identification of a novel species in the present study, closely related to *E. toletana* and *E. iniecta* provides further evidence for their transfer to a novel genus. “*E. beijingensis*” appears to be a close relative of the novel genus, but borderline AAI and POCP values and a contrasting FAME profile exclude its transfer to the novel genus. Further work is still required to determine the true taxonomic position of “*E. beijingensis*.”

The data generated in this study provides support, based on phylogenetic, genomic and phenotypic analyses, for the classification of a novel species and the reclassification of *E. toletana* and *E. iniecta* in a novel genus closely related to the genus *Erwinia*. Therefore, we propose to transfer *E. toletana* and *E. iniecta* to the novel genus *Winslowiella* gen. nov. as *Winslowiella toletana* comb. nov. and *Winslowiella iniecta* comb. nov., and the description of *Winslowiella arboricola* sp. nov. for the strains isolated from bleeding cankers of broadleaf hosts in Great Britain (type strain = BAC 15a-03b^T^ = LMG 32576^T^ = NCPPB 4696^T^).

### Description of *Winslowiella* gen. nov.

*Winslowiella* (Win.slow.i.el’la. N.L. fem. n. *Winslowiella*, named in honour of C.E. Winslow who proposed the genus name *Erwinia*).

The description is based on the data from Rojas et al. (2004); Campillo et al. (2015), and this study. Cells are Gram-negative, short, straight rods with rounded ends (0.5–1.5 × 1.5–3.5 μm) and can occur singly or in pairs and sometimes chains. Motile by peritrichous flagella. Catalase positive and oxidase negative, facultatively anaerobic. On LB agar colonies are slightly convex, circular with smooth edges, translucent, glistening, non-pigmented and cream to yellow in colour with a diameter of 1–3 mm. Strains can grow at 10–37°C, optimally at 28–30°C but not above 39°C, within the pH range of 6–8 and can tolerate supplemented salt conditions of up to 4%, with some species able to grow in 7% NaCl. Negative for arginine dihydrolase, lysine decarboxylase, ornithine decarboxylase, citrate utilisation, H_2_S production, urease, indole production, and gelatinase. β-galactosidase, tryptophan deaminase activity, acetoin production, and nitrate reduction are all variable.

Major fatty acids are C_16:0_, C_18:1_ ω7*c*, and summed feature 3 (C_16:1_ ω7*c* and/or C_16:1_ ω6*c*).

*Winslowiella* species are secondary invaders on diseased olive trees, and have been isolated from wheat aphids, bleeding cankers of broadleaf hosts and the wider environment.

The DNA G + C content ranges from 51.1 to 53.6 mol%. The type species is *Winslowiella toletana*.

### Description of *Winslowiella toletana* comb. nov.

*Winslowiella toletana* (to.le.ta.na. N.L. fem. adj. *toletana*, from Toletum, the Roman name for Toledo, the location from which the organisms were isolated).

Basonym: *Erwinia toletana* (Rojas et al., 2004).

The description is as given above for the genus and *E. toletana* in Rojas et al. (2004).

The DNA G + C content is 53.6 mol%.

The type strain is A37^T^ (=CFBP 6631^T^ = ATCC 700880^T^ = CECT 5263^T^) and was isolated from olive knots in association with *Pseudomonas savastanoi* pv. *savastanoi* as secondary invaders on diseased plants.

### Description of *Winslowiella iniecta* comb. nov.

*Winslowiella iniecta* (in.iec’ta. L. fem. part. adj. *iniecta*, thrust in, injected, referring to the fact that the first strains isolated from artificial media were introduced or “thrust in” *via* the aphid stylet).

Basonym: *Erwinia iniecta* (Campillo et al., 2015).

The description is as given above for the genus and *E. iniecta* in Campillo et al. (2015).

The DNA G + C content ranges from 51.1 to 52.2 mol%.

The type strain is B120^T^ (=CFBP 8182^T^ = NCCB 100485^T^), and was isolated from artificial diets fed on by *Diuraphis noxia*, biotype 2.

### Description of *Winslowiella arboricola* sp. nov.

*Winslowiella arboricola* (ar.bo.ri’cola. L. fem. n. *arbor*, a tree; L. masc./fem. n. suff. -*cola*, dweller; from L. masc./fem. n. *incola*, dweller; N.L. fem. n. *arboricola*, tree dweller).

The description is as given for the genus with the following additional characteristics. Strains can tolerate supplemented salt conditions of up to 7%. β-galactosidase activity is variable among strains, but tryptophan deaminase activity, acetoin production, and nitrate reduction are all negative. Acid is produced from L-arabinose, D-ribose, D-xylose, D-galactose, D-glucose, D-fructose, D-mannose, L-rhamnose, inositol, D-mannitol, *N*-acetyl glucosamine, arbutin, esculin ferric citrate, salicin, D-cellobiose, D-maltose, D-lactose, D-melibiose, D-trehalose, gentiobiose, and D-arabitol (API 50 CHB/E).

The following carbon sources are utilised in addition to those listed in Supplementary Table 4: β-methyl-D-glucoside, *N*-acetyl neuraminic acid, L-rhamnose, inosine, and mucic acid.

The DNA G + C content ranges from 53.5 to 53.6 mol%.

The type strain is BAC 15a-03b^T^ = LMG 32576^T^ = NCPPB 4696^T^, and was isolated from the bleeding lesion of a *Platanus* x *hispanica* (London plane) tree in London, Great Britain.

## Data availability statement

The data presented in this study are deposited in GenBank/EMBL/DDBJ under the accession numbers: OP422451 (16S rRNA); OP414924-OP414931 (atpD); OP414932-OP414939 (gyrB); OP414940-OP414947 (infB); OP414950-OP414955 (rpoB); and JAODIL000000000-JAODIM000000000 (whole genome).

## Author contributions

CB was involved in the conceptualisation, data curation, formal analysis, investigation, methodology, validation, visualization, and writing of the work. SK, BC, and DM were involved in the data curation, investigation of the work, and reviewing and editing of the manuscript. DA and SD were responsible for funding acquisition and reviewing and editing of the manuscript. All authors contributed to the article and approved the submitted version.

## Footnotes

1
https://bacterialplantdiseases.uk/bac-stop/
