# Supplementary material for: Transfer of Erwinia toletana and Erwinia iniecta to a novel genus Winslowiella gen. nov. as Winslowiella toletana comb. nov. and Winslowiella iniecta comb. nov. and description of Winslowiella arboricola sp. nov., isolated from bleeding cankers on broadleaf hosts
Source: Front Microbiol. 2022 Nov 17;13:1063107. doi: 10.3389/fmicb.2022.1063107 (PMC9712802; doi:10.3389/fmicb.2022.1063107)

Supplementary Material

**Supplementary Table S1:** Strains of *Winslowiella arboricola* sp. nov. investigated in this study

| **Strain** | **Location** | **Year of isolation** | **Source** | ***gyrB**** | ***rpoB**** | ***infB**** | ***atpD**** |
| --- | --- | --- | --- | --- | --- | --- | --- |
| BAC 15a-03b^T^ | St James Park, United Kingdom | 2020 | *Platanus x acerifolia,* bleeding lesion | OP414932 | OP414948 | OP414940 | OP414924 |
| BAC 1 -01-01 | St James Park, United Kingdom | 2020 | *Platanus x acerifolia,* bleeding lesion | OP414933 | OP414949 | OP414941 | OP414925 |
| Til 1 | Tidworth Garrison, United Kingdom | 2019 | *Tilia* x *europaea*, swab from bleeding lesion | OP414934 | OP414950 | OP414942 | OP414926 |
| Til 2 | Tidworth Garrison, United Kingdom | 2019 | *Tilia* x *europaea*, swab from bleeding lesion | OP414935 | OP414951 | OP414943 | OP414927 |
| Til 3 | Tidworth Garrison, United Kingdom | 2019 | *Tilia* x *europaea*, swab from bleeding lesion | OP414936 | OP414952 | OP414944 | OP414928 |
| Til 4 | Tidworth Garrison, United Kingdom | 2019 | *Tilia* x *europaea*, swab from bleeding lesion | OP414937 | OP414953 | OP414945 | OP414929 |
| Til 5 | Tidworth Garrison, United Kingdom | 2019 | *Tilia* x *europaea*, swab from bleeding lesion | OP414938 | OP414954 | OP414946 | OP414930 |
| Til 6 | Tidworth Garrison, United Kingdom | 2019 | *Tilia* x *europaea*, swab from bleeding lesion | OP414939 | OP414955 | OP414947 | OP414931 |

^T^ = type strain

* GenBank accession numbers

**Supplementary Table S2:** Whole genome sequence information of strains of *Winslowiella arboricola* sp. nov. investigated in this study

| **Strain** | **Genbank assembly accession** | **Biosample** | **Size**  **(Mbp)** | **Number of contigs (with PEGs)** | **N50** | **Number of coding sequences** | **Number of RNAs** | **GC content**  **(mol %)** |
| --- | --- | --- | --- | --- | --- | --- | --- | --- |
| BAC 15a-03b^T^ | GCA_025527015 | SAMN30788585 | 5.23 | 62 | 604 177 | 5 041 | 91 | 53.6 |
| Til 1 | GCA_025527035 | SAMN30788586 | 5.31 | 109 | 137 525 | 5 130 | 85 | 53.5 |

Supplementary Table S3: Average amino acid identity (AAI) values (bottom right) and percentage of conserved proteins (POCP) (top left) between *Winslowiella arboricola* sp. nov., *Winslowiella* *iniecta* comb. nov., *Winslowiella toletana* comb. nov. and existing species of the genus *Erwinia*

|  | **POCP** |  |  |  |  |  |  |  |  |  |  |  |  |
| --- | --- | --- | --- | --- | --- | --- | --- | --- | --- | --- | --- | --- | --- |
| **AAI** | **1** | **2** | **3** | **4** | **5** | **6** | **7** | **8** | **9** | **10** | **11** | **12** | **13** |
| **1** | 100 | 99 | 76 | 87 | 68 | 65 | 72 | 69 | 73 | 67 | 64 | 64 | 67 |
| **2** | 100 | 100 | 75 | 87 | 68 | 65 | 72 | 69 | 73 | 67 | 64 | 64 | 67 |
| **3** | 86 | 86 | 100 | 75 | 70 | 69 | 73 | 67 | 73 | 70 | 64 | 66 | 69 |
| **4** | 95 | 95 | 86 | 100 | 69 | 66 | 73 | 71 | 73 | 68 | 65 | 65 | 67 |
| **5** | 81 | 81 | 82 | 81 | 100 | 68 | 70 | 68 | 70 | 69 | 66 | 67 | 67 |
| **6** | 77 | 77 | 78 | 77 | 77 | 100 | 72 | 65 | 71 | 83 | 69 | 86 | 82 |
| **7** | 78 | 78 | 79 | 79 | 78 | 84 | 100 | 68 | 83 | 73 | 67 | 70 | 73 |
| **8** | 77 | 77 | 77 | 78 | 77 | 78 | 79 | 100 | 68 | 68 | 67 | 66 | 65 |
| **9** | 78 | 78 | 79 | 78 | 78 | 84 | 87 | 78 | 100 | 72 | 66 | 69 | 72 |
| **10** | 77 | 77 | 78 | 77 | 77 | 89 | 84 | 77 | 83 | 100 | 70 | 82 | 87 |
| **11** | 76 | 76 | 76 | 76 | 76 | 77 | 78 | 78 | 78 | 77 | 100 | 68 | 68 |
| **12** | 77 | 76 | 77 | 77 | 76 | 93 | 84 | 77 | 84 | 90 | 76 | 100 | 80 |
| **13** | 77 | 77 | 77 | 77 | 76 | 88 | 84 | 77 | 83 | 92 | 76 | 89 | 100 |

1 = *Winslowiella arboricola* BAC 15a-03b^T^ (GCA_025527015), 2 = *Winslowiella arboricola* Til 1 (GCA_025527035), 3 = *Winslowiella iniecta* B120^T^ (GCA_001267535), 4 = *Winslowiella toletana* DAPP-PG 735 (GCA_000336255), 5 = ‘*Erwinia beijingensis*’ LMG 27579^T^ (GCA_004022165), 6 = *Erwinia amylovora* ATCC 15580^T^ (GCA_017161565), 7 = *Erwinia aphidicola* JCM 21238 ^T^ (GCA_014773485), 8 = *Erwinia oleae* DAPP-PG531^T^ (GCA_000770305), 9 = *Erwinia persicina* NBRC 102418 ^T^ (GCA_001571305), 10 = *Erwinia piriflorinigrans* CFBP 5888^T^ (GCA_001050515), 11 = *Erwinia psidii* IBSBF 435^T^ (GCF_003846135), 12 = *Erwinia pyrifoliae* DSM 12163^T^ (GCA_000026985), 13 = *Erwinia tasmaniensis* ET1/99^T^ (GCA_000026185). ^T^ = type strain.

Supplementary Table S4: Positive phenotypic characteristics shared by current members of the genus *Winslowiella*

1 = *Winslowiella arboricola* (*n* = 3), 2 = *Winslowiella toletana* LMG 24162^T^ and 3 = *Winslowiella iniecta* (*n* = 4). Data for 3 taken from the literature (Campillo et al., 2015) . *n* = number of strains.

| **Characteristic** | **1** | **2** | **3** |
| --- | --- | --- | --- |
| **Utilisation of (Biolog):** |  |  |  |
| dextrin | + | + | + |
| D-maltose | + | + | + |
| D-cellobiose | + | + | + |
| gentiobiose | + | + | + |
| D-melibiose | + | + | + |
| α-D-glucose | + | + | + |
| D-mannose | + | + | + |
| D-fructose | + | + | + |
| D-galactose | + | + | + |
| D-mannitol | + | + | + |
| myo-inositol | + | + | + |
| glycerol | + | + | + |
| D-glucose-6-phosphate | + | + | + |
| L-alanine | + | + | + |
| L-aspartic acid | + | + | + |
| L-glutamic acid | + | + | + |
| L-serine | + | + | + |
| L-galactonic acid lactone | + | + | + |
| D-gluconic acid | + | + | + |
| D-glucuronic acid | + | + | + |
| glucuronamide | + | + | + |
| D-saccharic acid | + | + | + |
| acetic acid | + | + | + |
| **Variable reactions to (Biolog):** |  |  |  |
| D-trehalose | v |  | + |
| sucrose |  |  | + |
| D-raffinose | v | + | + |
| α-D-lactose | v | + | + |
| β-methyl-D-glucoside | + |  | + |
| *N*-acetyl-D-glucosamine | v |  | + |
| *N*-acetyl neuraminic acid | + | + |  |
| L-fucose | v | + |  |
| L-rhamnose | + |  | + |
| inosine | + | + |  |
| D-sorbitol |  |  | + |
| D-serine |  | + |  |
| L-arginine | v | + | + |
| L-pyroglutamic acid |  |  | + |
| bromo-succinic acid | v | + | + |
| formic acid | v | + |  |

+, 90 – 100 % strains +; v, variable;

**Supplementary Table S5:** Notable virulence genes identified computationally from species of *Winslowiella* gen. nov. Type strains are listed with the alignments made from the comparison of the proteome to the VFDB indicating their pathogenic potential; the genes identified in plant bacterial interactions via PIFAR which indicate how the strains can interact with plants and predicted T3SS effectors from Effectidor.

| **Virulence genes** | ***Winslowiella arboricola***  **BAC 15a-03b^T^** | ***Winslowiella toletana***  **DAPP-PG 735** | ***Winslowiella iniecta***  **B120^T^** | ***‘Erwinia beijingensis*’**  **LMG 27579^T^** |
| --- | --- | --- | --- | --- |
| Biofilm | 4 | 4 | 7 | 4 |
| Effector delivery system | 16 | 17 | 35 | 16 |
| Nutritional/Metabolic factors | 28 | 16 | 35 | 3 |
| Immune modulation | 41 | 35 | 34 | 35 |
| Response regulation | 6 | 6 | 6 | 3 |
| Motility | 54 | 57 | 58 | 57 |
| Stress survival | 2 | 3 | 1 | 3 |
| Invasion | 1 | 1 | 1 | 2 |
| Adherence | 5 | 6 | 9 | 9 |
| Isocitratelyase | 2 | 2 | 2 | 0 |
| Antimicrobial activity/Competitive advantage | 5 | 6 | 4 | 4 |
| **‘Plant only interaction’ genes** |  |  |  |  |
| Detoxification | 8 | 8 | 8 | 8 |
| EPS | 4 | 4 | 4 | 4 |
| LPS | 5 | 5 | 5 | 5 |
| PCWDE | 9 | 9 | 9 | 9 |
| Toxins | 6 | 6 | 6 | 6 |
| **T3SS predictions** |  |  |  |  |
| Core genes | 28 | 27 | 41 | 16 |
| Effectors | 6 | 5 | 4 | 8 |
| Potential novel effectors ‘High confidence’ RC ≥ 0.5 | 1 | 4 | 2 | 2 |
| Potential novel effectors ‘Low confidence’ 0.3 ≤ RC ≤ 0.5 | 1 | 1 | 0 | 21 |

Supplementary Figure S1: Maximum likelihood tree based on the almost complete 16S rRNA gene sequences (1,346 bp) of *Winslowiella* gen. nov., *Winslowiella arboricola* sp. nov. and the closest phylogenetic relatives. Bootstrap support values higher than 50 % after 1000 replicates are shown next to the branches and *Plesiomonas shigelloides* was used as an outgroup. The fraction of substitutions per site is indicated by the scale bar.


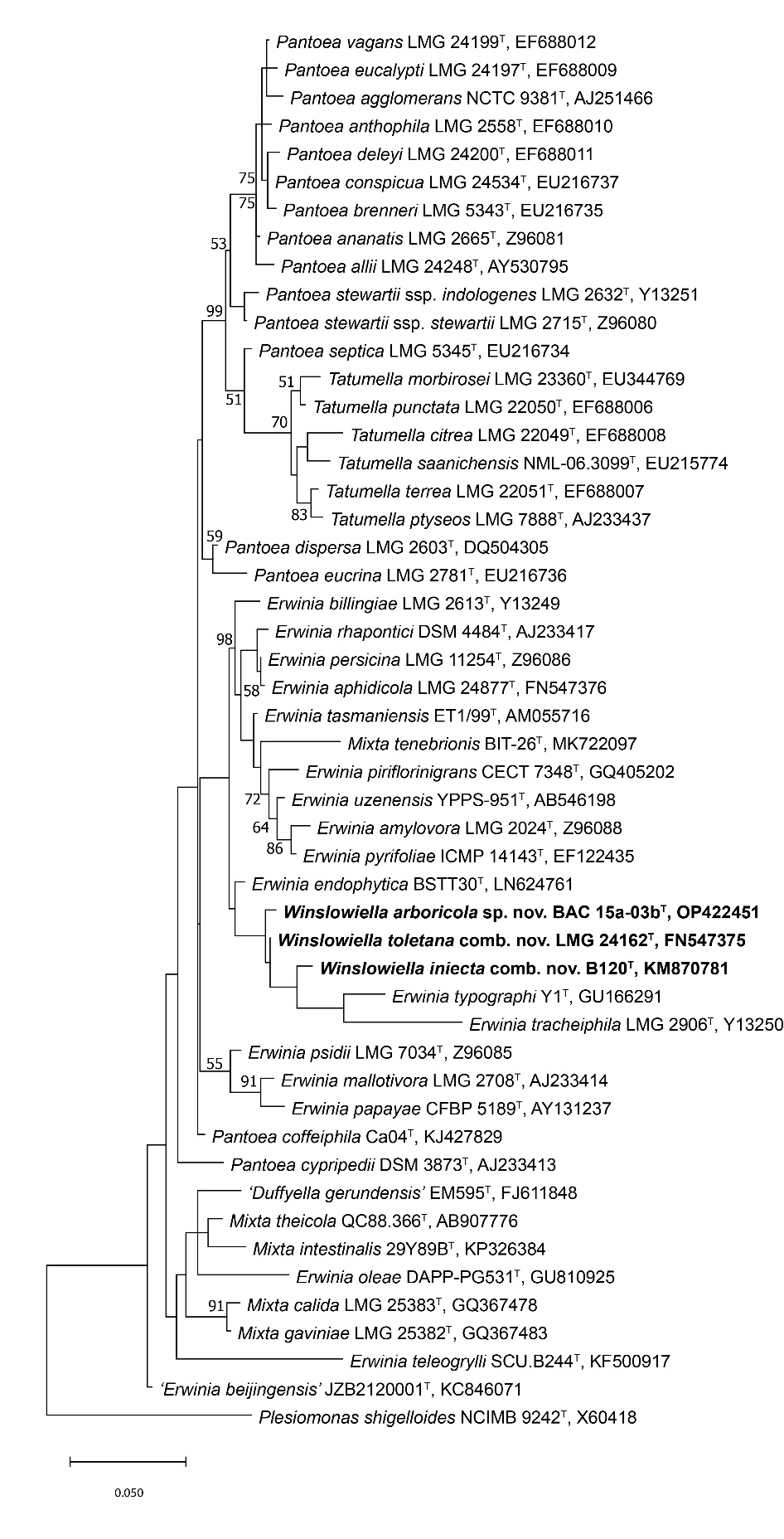


**Supplementary Figure S2**: BOX-PCR fingerprinting band patterns of *Winslowiella aboricola* sp. nov. strains. Lanes: (1) BAC 1-01-01, (2) BAC 15a-03b^T^, (3) Til 1, (4) Til 2, (5) Til 3, (6) Til 5, (7) Til 6, (8) negative control. Hyperladder 1kb (Bioline)


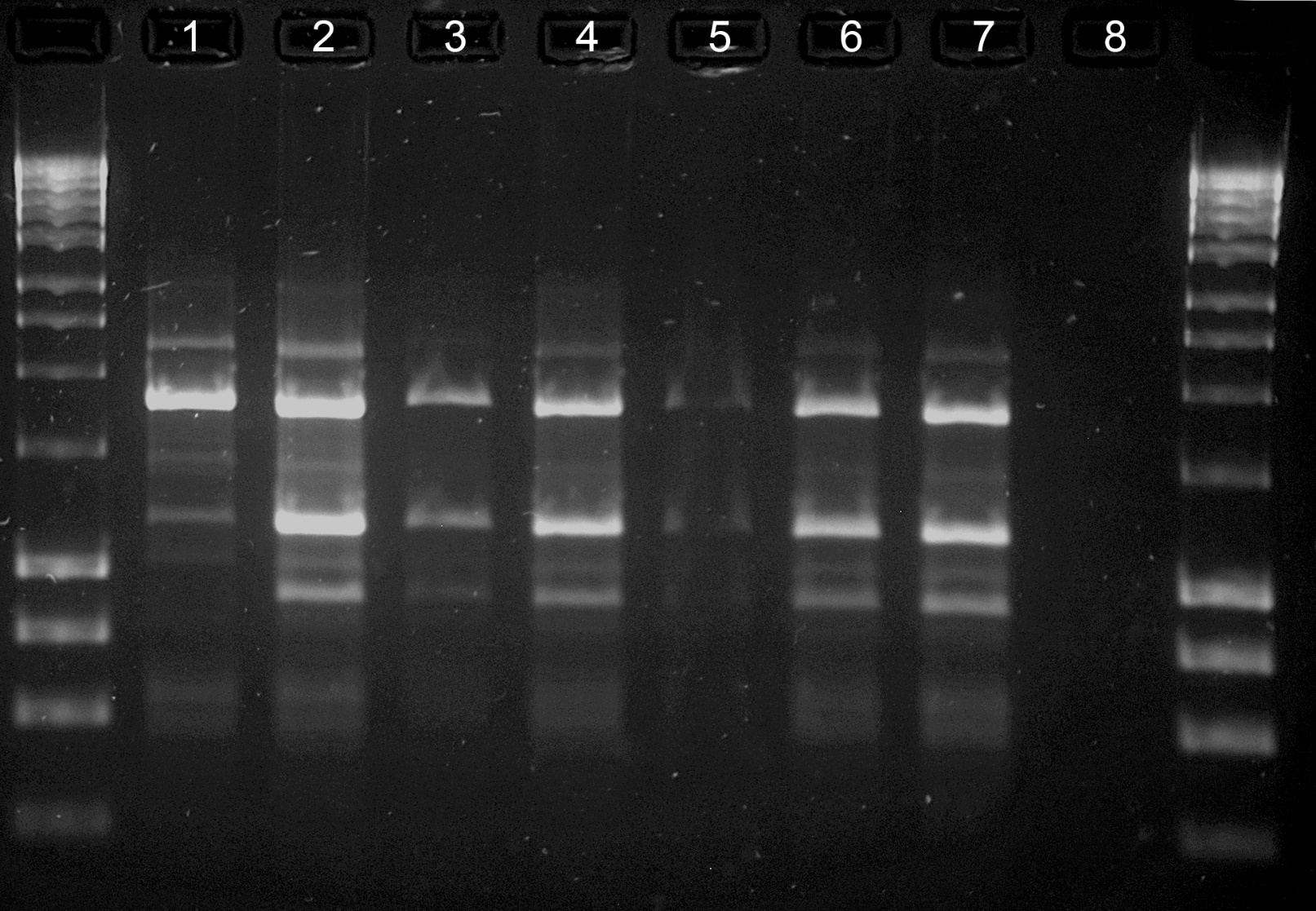


**Supplementary Figure S3**: Transmission electron microscopy of *Winslowiella aboricola* sp. nov. BAC 15a-03b^T^ displaying the peritrichous flagella arrangement. Scale bar, 1 µm.


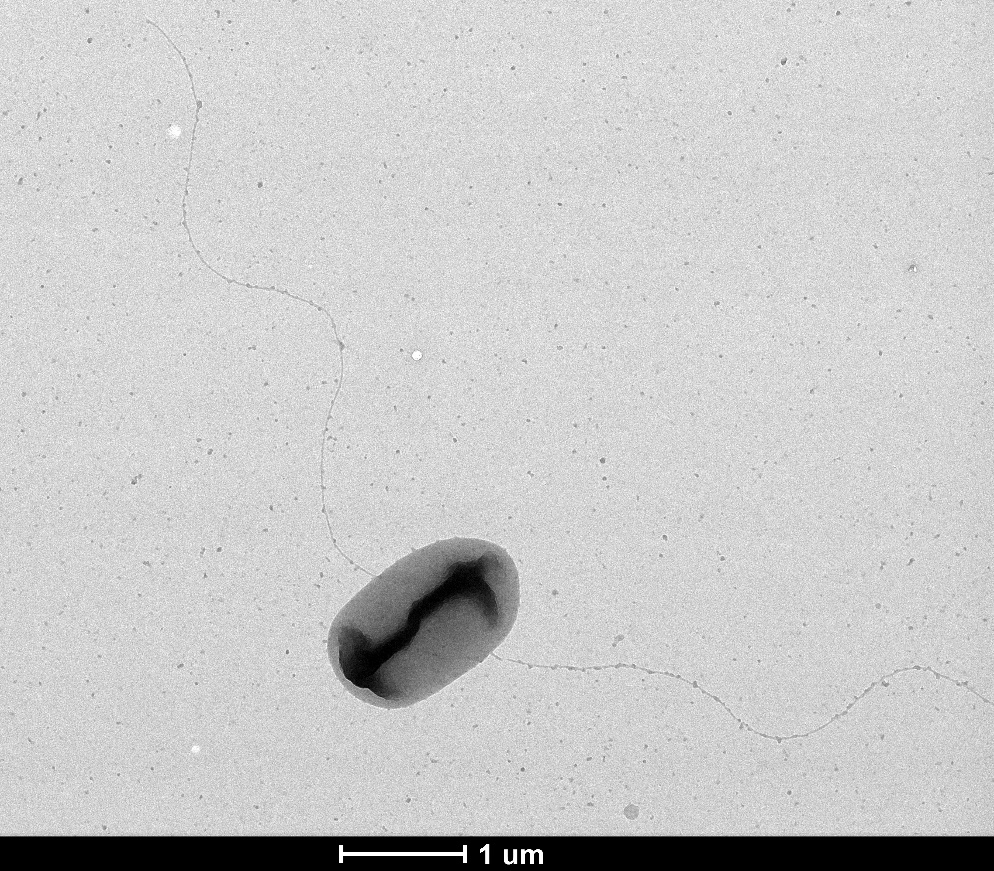

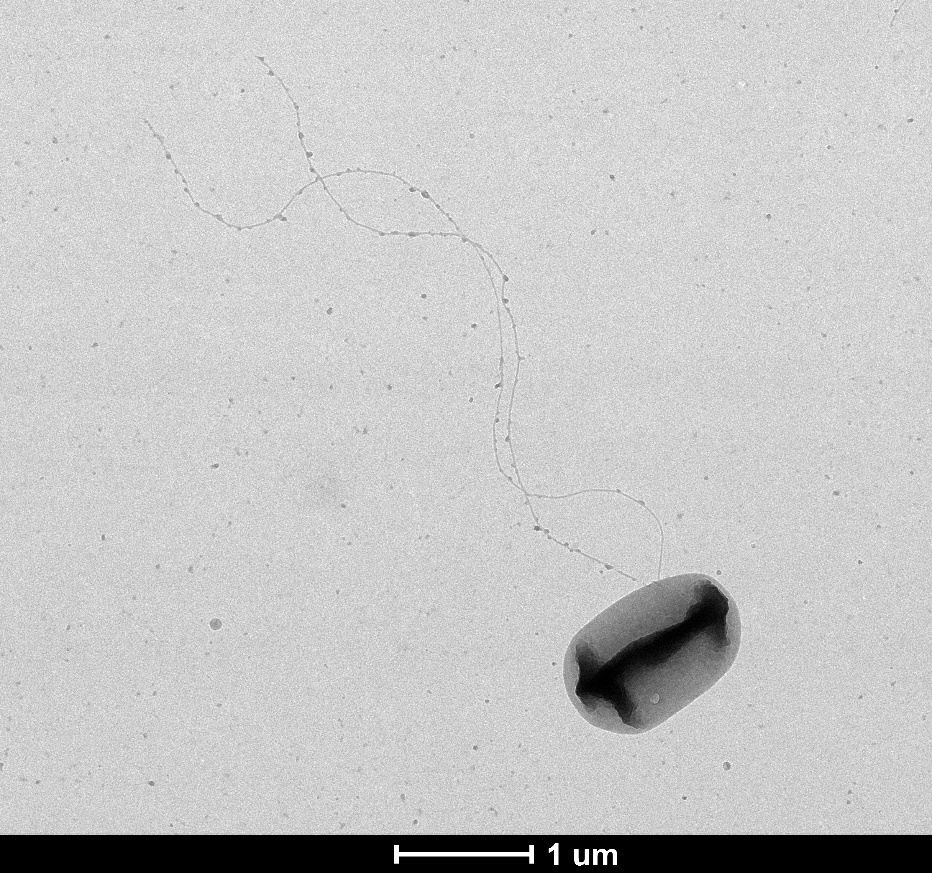

Supplement: Supplementary file 1 [file Data_Sheet_1.docx]
